# Death by Segregation: Does the Dimension of Racial Segregation Matter?

**DOI:** 10.1371/journal.pone.0138489

**Published:** 2015-09-23

**Authors:** Tse-Chuan Yang, Stephen A. Matthews

**Affiliations:** 1 Department of Sociology, Center for Social and Demographic Analysis, University at Albany, State University of New York, Albany, NY, United States of America; 2 Departments of Sociology and Anthropology, Population Research Institute, Pennsylvania State University, University Park, PA, United States of America; Johns Hopkins University, UNITED STATES

## Abstract

The county-level geographic mortality differentials have persisted in the past four decades in the United States (US). Though several socioeconomic factors (e.g., inequality) partially explain this phenomenon, the role of race/ethnic segregation, in general, and the different dimensions of segregation, more specifically, has been underexplored. Focusing on all-cause age-sex standardized US county-level mortality (2004–2008), this study has two substantive goals: (1) to understand whether segregation is a determinant of mortality and if yes, how the relationship between segregation and mortality varies by racial/ethnic dyads (e.g., white/black), and (2) to explore whether different dimensions of segregation (i.e., evenness, exposure, concentration, centralization, and clustering) are associated with mortality. A third goal is methodological: to assess whether spatial autocorrelation influences our understanding of the associations between the dimensions of segregation and mortality. Race/ethnic segregation was found to contribute to the geographic mortality disparities. Moreover, the relationship with mortality differed by both race/ethnic group and the dimension of segregation. Specifically, white/black segregation is positively related to mortality, whereas the segregation between whites and non-black minorities is negatively associated with mortality. Among the five dimensions of segregation, evenness and exposure are more strongly related to mortality than other dimensions. Spatial filtering approaches also identified six unique spatial patterns that significantly affect the spatial distribution of mortality. These patterns offer possible insights that help identify omitted variables related to the persistent patterning of mortality in the US.

## Introduction

While the United States (US) as a whole has experienced a significant decrease in mortality since World War II from approximately 20 deaths per 1,000 population to 8 deaths per 1,000 population [1], two mortality patterns have remained–racial/ethnic and geographic mortality disparity. An example of the racial/ethnic mortality disparity can be found in data comparing the mortality of non-Hispanic blacks to those of other race/ethnicity groups. In 2011, the age-adjusted mortality among non-Hispanic blacks was 9.04 deaths (per 1,000 population), whereas non-Hispanic whites and Hispanics had 7.54 deaths and 5.39 deaths, respectively [2]. Similarly, geographic mortality disparities across the US has been stable for the past four decades [3]. For example, the counties in the Black Belt and lower Mississippi Valley have had relatively high mortality rates, whereas those in the Great Plains, Mid-West, and along the US/Mexico border much lower [3]. Explanations for these geographic mortality disparities have mainly focused on socioeconomic factors (e.g., poverty, unemployment, educational attainment, income inequality, and social capital), demographic structure (e.g., racial compositions), and context (e.g., rurality) [4–9]. While these factors are important, the variation in mortality along with race/ethnicity and geographic dimension has not been fully explained in the literature. A potential determinant, namely race/ethnic segregation and the different dimensions of segregation, has–perhaps surprisingly–rarely been incorporated into ecological mortality research, particularly nationwide county-level studies [10].

The concept of segregation is complex but earlier studies on health and segregation largely overlook the complexity of how to measure segregation. Massey and Denton [11] have shown that segregation can be classified into five different dimensions–*evenness*, *exposure*, *concentration*, *centralization*, and *clustering*–and each of these dimensions can in turn be calculated in multiple ways. Little attention has been paid to the nuances of race/ethnic segregation in health research [12, 13]. A recent study used three different measures of segregation to explore the associations with mortality [14]; however, this analysis focused solely on white/black segregation, ignoring other race/ethnic groups. In this context, this paper makes a unique contribution to the literature by examining whether racial/ethnic segregation and specific dimensions of segregation are associated with US county-level mortality. The findings of this study will substantiate the utility of ecological mortality research, and contribute new material and perspectives on discussions of segregation and health.

Research on race/ethnic segregation has tended to use the metropolitan and nonmetropolitan definitions proposed by the Office of Management and Budget and to focus on US metropolitan areas [15–17]. This metropolitan focus persists even though race/ethnic segregation has been reported to be higher in nonmetropolitan areas where the racial composition is generally less diverse [18–21]. Following Lobao and colleagues [22], we adopt a county-level perspective to consider both metropolitan and nonmetropolitan areas of the US and this approach complements the metropolitan focus in the literature in the following ways. First, the metropolitan focus is often on a single or a subset of metropolitan area(s) and does not cover the entire US; with the consequence that nonmetropolitan areas are overlooked. The county-level perspective provides a nationwide assessment of the relationship between segregation and mortality and allows for a direct comparison with previous county-level studies, e.g., Cossman et al [3]. Second, some large metropolitan areas are comprised on multiple counties and in this context the findings are specific to a metropolitan and not for each county within a metropolitan area; the consequence here being that conventional approaches overlook the heterogeneity within metropolitan areas and may obscure the variation in mortality within a metropolitan area [10]. Thus the county-level perspective disentangles the segregation-mortality relationship within a metropolitan area and offers nuanced insight into the geographic mortality differential across the US. However, the use of county-level data in an ecological analysis can also raise several methodological issues. Most specifically, counties are not independent observations and thus using county-level data without controlling for spatial dependence may result in biased estimates and lead to invalid or misleading interpretation of findings [23]. In this paper, we directly address this issue via the use of spatial filtering regression methods; an emergent technique in spatial analysis.

This study has four main goals: (1) to understand whether race/ethnic segregation is a determinant of mortality in US counties, (2) to investigate whether the relationship between segregation and mortality varies by individual minority groups (i.e., blacks, Hispanics, and Asians/Pacific Islander) from the dominant group (i.e., whites), (3) to explore whether different dimensions of segregation are associated with mortality, and finally (4), a methodologically informed goal, to assess whether spatial dependence influences our understanding of the associations between the individual dimension of segregation and mortality. Our analysis focuses on US county-level mortality data for 2004–2008.

## Segregation and Health by Race/Ethnicity

The conventional belief that race/ethnic segregation is adversely related to health is partially rooted in the ethnic stratification perspective. Collins and Williams argued [24] that race/ethnic segregation could be understood as a structural manifestation of racism against minorities, and in particular, non-Hispanic blacks (hereafter blacks). Discrimination against minority groups can take on many forms. Among the most dominant forms is when discrimination fuels the residential sorting process, which as Logan [25] noted, is a powerful mechanism maintaining the advantages of the majority group and generates ethnic stratification. From this perspective, people living in racially segregated neighborhoods are exposed to multiple health risk factors, such as poverty, crimes, and poor public services [24, 26], and these risk factors are associated with poor health and racial health disparities [27]. The legal challenges to discrimination and discriminatory practices enacted since the 1960s has in part lead to the decline in white/black segregation [28].

Extending the ethnic stratification perspective, there are four key mechanisms underlying the common belief that race/ethnic segregation is detrimental to health: (1) areas with high levels of race/ethnic segregation have poor socioeconomic status (e.g., high poverty and high unemployment), which may contribute to poor health outcomes [24, 26, 29]; (2) race/ethnic segregation is associated with political alienation and powerlessness and these factors may lead to relatively few resources being channeled into a minority area; (3) the environment of an area with high race/ethnic segregation is more likely to be neglected and lacking infrastructure [30, 31]; and (4) the hospitals and community health care centers in highly segregated areas have been found to lack state-of-the-art technology or facilities that can reduce mortality and living in segregated areas may be translated into poor access to and/or utilization of health care services [32, 33]. These pathways, individually and in combination, may expose local residents to multiple health risks; a negative association between race/ethnic segregation and health is expected [27]. The conventional framework is heavily driven by the ethnic stratification perspective, but this framework may not be applicable to non-black minority groups, such as Hispanics and Asians/Pacific Islanders.

The landscape of racial composition has rapidly changed since the 1980s mainly due to the influx of immigrants from both Latin America and Asia [34]. The geographically mixing and thus the race/ethnic segregation of Hispanics and Asians/Pacific Islanders with whites have been transformed [35, 36]. Researchers examining the residential sorting processes for different minority groups find differences between the growing race/ethnic groups and the white population compared to the processes accounting for white/black segregation. Though Asian immigrants have stronger social capital and higher educational attainment than Hispanic immigrants, both groups tend to live in an ethnically bound neighborhood or enclaves [37]. This living arrangement can help them to improve their socioeconomic situation and the process of adaption to the new society. That is, race/ethnic segregation between Hispanics and Asians from whites may be strategic for these minority groups. Logan and colleagues [37] identified two types of neighborhood that serves the goal to help immigrants to survive and thrive: ethnic enclaves and ethnic communities. The distinction between these two neighborhoods is grounded in the motives of minority residents. Specifically, the former plays a temporary or transitional role in the process of adaption, whereas the latter is established by minority members who voluntarily live nearby, usually in the later stage of the process of adaption [37]. Despite the difference, the shared and imperative function of both neighborhoods is to help minorities to thrive or accumulate social and financial capital. This function may encourage Hispanics and Asians to be self-segregated to take advantage of ethnically bound neighborhoods. Racial segregation may, hence, be beneficial to Hispanics and Asians for the following reasons.

First, living in an ethnic enclave/community can translate into increased social support, frequent social engagement with people of the same race/ethnicity, and fewer challenges emerging from linguistic isolation [31]. These factors foster strong social cohesion that may facilitate health and well-being [38]. Second, and related, an ethnic enclave/community may provide social, economic, and structural resources generated by the close-knit social connections or among residents of the same race/ethnicity [39, 40]. That is, the access to educational, information, and occupational opportunities in an ethnic enclave/community may be better in an ethnic enclave or community than in other types of neighborhood. Third, being segregated from the dominant racial group indicates a low level of exposure to direct racial discrimination, and in such a neighborhood, the norm that racial discrimination is intolerable would prevail thanks to a strong ethnic identity [41, 42]. All of these factors lead us to argue that race/ethnic segregation may be beneficial to health (i.e., mortality in this study), particularly for non-black minorities. It should be noted that scholars recently found the living in ethnic enclaves was associated with no access to psychiatrist in a neighborhood, which conflicts with our arguments [33], however, the majority of the literature still provides support to the aforementioned benefits.

As all-cause mortality is directly affected by the prevalence of diseases and/or health behaviors, in order to better estimate the relationship between mortality and segregation, it becomes necessary to control for population health in a county. Somewhat surprisingly, relatively few ecological mortality studies in the past decade took this aspect into consideration, *cf*. Kindig and Cheng [43]. This study will take population health measures into account. Should this approach be used and the relationship between segregation and mortality remain, we obtain strong evidence to support our substantive arguments.

Since segregation and mortality are essentially ecological measures, their distribution across counties may be spatially dependent as are many other social data [44]. To obtain unbiased estimates, researchers have become increasingly aware of the importance to take spatial dependence into account, particularly in ecological demographic studies [45]. We will discuss how this study addresses this methodological issue later.

## Why Do Dimensions of Segregation Matter?

We argue that the aforementioned mechanisms linking segregation and health may be better captured by some specific dimensions of segregation and we propose two reasons to justify this. First, it has been found that the levels of segregation are not necessarily consistent across the five dimensions. For example, Wilkes and Iceland [17] examined the five dimensions of segregation among the US metropolitan areas and found that the cities with high concentration and centralization tend to have relatively low evenness, exposure, and clustering. That is, for a given metropolitan area, a highly uneven distribution of minority groups does not necessarily translate into a high concentration of minority groups. In this way, using a single dimension of segregation may not fully capture the mechanisms from which segregation affects health.

Second, the social and health implications differ by the dimensions of segregation. For example, exposure focuses on the potential contact between the minority and dominant racial/ethnic groups and high levels of contact indicate less segregation. The exposure dimension better echoes the concept of social integration or social capital that facilitates population health [38, 46] than do other dimensions. In contrast, evenness is a measure of the spatial distribution of minority groups; however, the evenness dimension may be less relevant to health or social outcomes than clustering as high clustering indicates a racial/ethnic enclave, where the life chances may be better/worse than other types of communities [37]. Similarly, higher centralization has been found to increase the odds of an area being a primary care physicians shortage areas, particularly for blacks [32], indicating that living in a highly centralized area would lead to the lack of access to primary care physicians. Given these potential differences across dimensions, it is important to examine the relationship between health and the dimensions of segregation.

## Using Spatial Structure to Address Spatial Dependence

In this study, mortality and segregation both are measured for ecologic units (i.e., counties) and the spatial relationships between these units need to be explicitly incorporated into an analytic strategy if we are to obtain unbiased estimates of the relationships that exist between them [47, 48]. Several demographers have explicitly incorporated a spatial perspective into mortality research based on a spatial econometric framework and concluded that applying a spatial perspective to county-level data would generate better model fits and more accurate predications than the models without a spatial component [8, 9, 14]. Arguably, spatial econometric methods are the most popular in spatial demography but they can only provide an *overall* assessment of *how much* spatial structure matters. A common criticism of the spatial econometrics approach is that researchers do not know *how* spatial structure matters. Griffith [49, 50] and Tiefelsdorf and Griffith [51] have proposed spatial filtering methods that use eigenfunctions to create a series of spatial patterns that are mutually unrelated but associated with the spatial structure underlying the spatial units. A recent study by Thayn and Simanis [52] suggested that the spatial filtering approach effectively minimizes spatial misspecification errors, improves model fit, and eliminates spatial dependence. In addition, the unrelated spatial patterns can be visualized (i.e., mapped) to gain further insight into how spatial structure contributes to the analysis and potentially shed new light on omitted variable bias [49]. Indeed, perhaps the most distinctive feature of the spatial filtering approach lies in the decomposition of errors and that allows for the visualization of unknown spatial processes that affect the spatial pattern of the dependent variable (i.e., mortality). To our knowledge, our study is the first to use a spatial filtering approach to examine the mortality pattern across US counties.

Drawing from the discussion above, we propose two substantive research hypotheses and one derived from our use of spatial filtering. The main substantive hypotheses are:

1. White/black segregation is positively related to mortality as the segregation process (ethnic stratification) is rooted in discrimination, and the segregation between whites and non-black minority groups are beneficial to mortality as the segregation processes lead to the formations of enclaves and/or communities.
2. Not all five dimensions of segregation matter and the exposure dimension is the most relevant as dimension as it captures the mechanisms linking segregation and mortality by assessing the level of interactions between the minority and the dominant racial/ethnic group.

We also expect that

1. spatial dependence will bias the estimates of the relationships between segregation and mortality and as such a spatial filtering approach can help refine our model specification and be used to identify the spatial patterns. We will examine these issues focusing on five dimensions of segregation.

## Data and Measures

The county-level mortality rate is the dependent variable of this study. Based on the Compressed Mortality Files maintained by the National Center for Health Statistics [53], we created the 2004–2008 five-year average mortality rates that are standardized to the 2006 US age-sex population structure. Using the five-year average rates minimizes the fluctuations across years and this approach has been used in recent ecological mortality studies [54, 55]. As race/ethnic segregation plays a crucial role in this study, rather than standardize mortality rates with respect to racial groups, instead we include the race/ethnic composition of a county in the analysis.

While segregation is the key independent variable of this study, we further control for six factors that have been commonly included in ecological mortality research, namely metropolitan status, socioeconomic status, racial composition, income inequality, social capital, and population health. Segregation and each of the other six factors are measured with different variables; we discussed the operationalization of our analytical variables in detail below:

### Segregation

We measured all five dimensions of racial segregation. Specifically, the “evenness” and “exposure” dimensions are measured with *entropy* [56] and the *isolation index*, respectively. The entropy index assesses the average deviation of the sub-unit (i.e., tract in this study) from the county’s racial diversity, whereas the isolation index measures “the extent to which minority members are exposed only to one another (p.288)” [11]. Entropy and isolation index both range between 0 and 1 and higher values of entropy and isolation suggest higher segregation. The “centralization” dimension is captured with the *absolute centralization index* that is developed to understand if the minority group is distributed around the center of a county. The absolute centralization index varies between -1 and 1 where a positive value indicates that minorities tend to live nearer the center of a county, whereas the negative values suggest that minority populations live in the outlying areas [11]. The “concentration” dimension is based on the *delta index* [57] and is calculated to assess the proportion of minority members who live in the areas where the minority density is higher than average, ranging between 0 (no concentration) and 1. The delta index represents the proportion of a minority group that have to move to reach a uniform density within an area [11]. The fifth and final dimension, “clustering,” is measured by the spatial proximity index [58], which is the average of proximities within the minority and majority group, respectively. A spatial proximity index greater than 1 suggests that minority members live close to one another and so do the majority. When a spatial proximity index is less than 1, it means that majority and minority members live closer to each other than the members of their own groups.

We calculated the five segregation measures by the following three race/ethnicity combinations: non-Hispanic whites vs. non-Hispanic blacks (white/black), non-Hispanic white vs. Hispanic (white/Hispanic), and non-Hispanic white vs. Asians/Pacific Islanders (white/API). We only focused on the three largest minority groups in order to avoid unreliable segregation measures due to small number issues in many counties. The statistical procedures developed by Iceland and colleagues [35] were applied to the 2010 Census Summary File 1 race/ethnicity data and we aggregated tract data into counties to obtain the fifteen segregation measures for all US counties.

### Metropolitan

The rural-urban mortality differential in US counties has been documented and metropolitan counties have been found to have higher mortality rates than their rural counterparts [7]. Taking other socioeconomic covariates, such as poverty and educational attainment, into account does not fully explain the geographic mortality differential and thus it is important to consider metropolitan status in this study. We employed the *metropolitan status* developed by the US office of Management and Budget in 2010 to dichotomize US counties into metropolitan and nonmetropolitan counties. While the heterogeneity within each group could be great, a recent study [9] reported that the conclusions based on the metro-nonmetropolitan dichotomy were similar to those drawn from a finer metropolitan measure (i.e., the rural-urban continuum codes). That is, the dichotomous metropolitan status provides modeling parsimony to this study.

### Socioeconomic status

As discussed previously, socioeconomic status is an important factor for mortality. We will use 2005–2009 American Community Survey (ACS) to obtain a set of social indicators and apply principal component analysis to reduce the total number of variables. This variable reduction approach is comparable with that proposed by Sampson and colleagues [59]. Indeed, more specifically, the following four indicators loaded on the concept of *social affluence* (factor loading in parenthesis): the log of income per capita (0.72), the percentage of population aged 25 or over with at least a bachelor’s degree (0.91), the percentage of population working in professional, administrative, and managerial positions (0.87), and percentage of families with annual incomes higher than $75,000 (0.87). Similarly, the concept of *social disadvantage* was derived from three indicators: the poverty rate (0.72), the percentage of population receiving public assistance (0.71), and the percentage of female-head households with children below 18 (0.81). These two concepts–social affluence and social disadvantage–account for more than 70 percent of the variation among these seven indicators. We used the regression approach to generate the factor scores included in the analysis.

### Racial composition

As the dependent variable, county-level mortality rate, was not standardized with the race/ethnicity structure, we included racial composition variables namely *the proportion of blacks*, *the proportion of Hispanics*, *and the proportion of other races*. It should be noted that racial composition is highly correlated with the five segregation measures. For example, the proportion of blacks is strongly associated with the white/black isolation index (Pearson’s R = 0.90). To avoid multicollinearity, we excluded the racial composition variable from models that include the same group-specific minority segregation measures (e.g., excluding the proportion of blacks in the model with all five white/black segregation indices). Furthermore, even when the proportion of Asians or Pacific Islanders is separated from the proportion of other races and included in the analysis, its association with mortality was not statistically significant (due to vary small proportions across counties) and our conclusions were not altered. For the purpose of modeling parsimony, we just presented the results using the proportion of other races.

### Income inequality

The relationship between income inequality and health has drawn much attention in the literature [60] and income inequality is closely related to racial segregation [61]. To understand whether the association between segregation and mortality is independent of income inequality, we used the 2005–2009 ACS household income data to calculate the Gini coefficient and included it in the analysis to control for the level of income inequality in a county. The Gini coefficient ranges between 0 and 1 and a larger Gini coefficient indicates a higher level of income inequality. As the top-coded category in the ACS income data is an open category ($200,000 or above), the income inequality measure may be underestimated (a common drawback when calculating the Gini coefficient with grouped, instead of individual, income data).

### Social capital

As for social capital, we adopted *social capital index* developed by Rupasingha et al [62], measuring county-level social capital based on Putnam [63]. Four indicators were used to assess the strength of social capital in a county: the number of associations (e.g., sports clubs) per 10,000 population, the number of non-profit organizations per 10,000 population, the mail response rate for the decadal census, and the presidential election voting rate. Using principal factor analysis, Rupasingha and Goetz [64] calculated the 2005 social capital index (the latest available) for US counties and a larger social capital index suggests stronger social capital in a county. We used the 2005 social capital index in the analysis.

### Population health

As discussed previously, the county-level mortality rate is a consequence of overall population health in a county and including population health covariates helps us to better clarify the segregation-mortality association. Somewhat surprisingly, few previous studies explicitly include the population health measures of a county in the analysis. To fill this gap, we obtained two reliable population health measures from the University of Wisconsin Population Health Institute [65]: *average unhealthy days per month* of the population in a county and the *adult obesity rate*. The unhealthy days include both mental and physical unhealthy days based on residents’ answers to the question of “how many days during the past 30 days was your physical and mental health not good.” The adult obesity rate indicates the percentage of adults with a body mass index greater than 30. Both measures were originally developed by the Centers for Disease Control and Prevention (CDC) and have been included in the Behavioral Risk Factor Surveillance Surveys conducted and maintained by CDC. The reliability and validity of these measures have been examined by CDC and the University of Wisconsin Population Health Institute. The methodologies used to obtain these county-level health measures could be found elsewhere [66–68].

### Methodology: Spatial filtering

Mortality rates are not evenly distributed across the US [3] and importantly these patterns indicate strong spatial dependence. Based on a spatial filtering approach, the spatial pattern of mortality can be decomposed into three parts: (1) a spatial trend that can be explained by a set of independent variables related to mortality, (2) a spatial process that could only be captured by the factor that is not included as an independent variable, and (3) the random disturbances. The eigenvector spatial filtering approach aims to extract distinctive spatial patterns that are not only associated with the spatial process in (2) but also account for the spatial autocorrelation in the dependent variable (i.e., mortality). This eigenvector spatial filtering approach can be adopted by most classical regression models, such as the ordinary least squares (OLS) and logistic regression; the estimates of the spatially filtered models would be unbiased and the interpretations of the estimates remain the same [51].

Since the dependent variable of this study is continuous, we discuss the eigenvector spatial filtering approach under the OLS framework. The basic OLS regression model for mortality can be expressed as ***y*** = ***βX*** + ε*, where ***y*** is a vector of mortality rates, ***β*** represents the parameters associated with a set of independent variables, ***X***, and ε* are spatially autocorrelated errors. The eigenvector spatial filtering approach further decomposes ε* into ***Eγ* + *ε***, where ***E*** represents a set of unspecified factors that are related to the spatial autocorrelation of mortality, ***γ*** is a set of estimates for ***E*** (i.e., the relationships with mortality), and ***ε*** denotes the random disturbances. In empirical research, ***E*** is often, if not always, unknown. However, as the goal of spatial filtering is to account for spatial autocorrelation in the dependent variable, ***E*** can be created based on Moran’s I [49, 51], a commonly used measure of global spatial autocorrelation [69].

To obtain ***E***, we create a set of dummy variables (***B***) based on the equation below:

$$B=\left(I-11^{T}/n\right),$$ (1)

where ***I*** is an *n*-by-*n* identity matrix and **1** is a vector of length *n* containing ones. The superscripted *T* indicates a transposed matrix and *n* is the total number of observations. We can use ***B*** to transform the spatial weight matrix (***C***) underlying the spatial data:

$$\Omega =BCB=\left(I-11^{T}/n\right)C\left(I-11^{T}/n\right),$$ (2)

where **Ω** is the transformed matrix and ***C*** is the spatial weight matrix based on the spatial relationships (defined by adjacency or distance) among units. The fact that the eigenvectors of **Ω** are orthogonal (i.e., uncorrelated) is the reason why ***C*** has to be transformed. The orthogonal eigenvectors indicates the unique spatial patterns filtered from the spatially autocorrelated errors.

The Moran’s I value for each eigenvector given a specific spatial weight matrix (***C***) can be expressed as a function of the eigenvalues of **Ω** [49, 50]:

$${\text{Moran}}^{\text{'}}\text{s I}=(n/1^{T}C1)*eigenvalue\left(\Omega \right),$$ (3)

Eq (3) suggests that the Moran’s I values can be computed for any numerical values of a dependent variable (e.g., mortality) in a data set with *n* observations. It should also be noted that the first eigenvector (denoted as ***E***_**1**_) will have the largest Moran’s I value given the spatial structure ***C*** and the second eigenvector will be a set of numbers that will make Moran’s I statistic largest, yet smaller, than the Moran’s I of the first eigenvector. Similarly, the third eigenvector includes the real numbers that generate the largest Moran’s I value that is smaller than the Moran’s I of the second eigenvector. In this fashion, spatial dependence, as measured by the Moran’s I, decreases as the order of eigenvector increases [49].

In order to tie the spatial filtering procedures above to the OLS regression, one needs to incorporate a set of independent variables (***X***) into Eq (1) as follows:

$$B_{OLS}=M=I-X{\left(X^{T}X\right)}^{-1}X^{T},$$ (4)

where ***X*** is a matrix containing the independent variables, which is the same ***X*** included in the basic OLS regression. Multiplying ***M*** by ***y*** results in the matrix of ε* and the eigenvectors of the transformed matrix (***MCM*)** are hence derived from and are orthogonal to the independent variables (***X***). This ***MCM*** matrix still could be applied to Eq (3) and the spatial filtering procedures could be implemented. Importantly, the selected eigenvectors based on Moran’s I can be added to the OLS model as supplementary covariates that mainly account for spatial autocorrelation in the dependent variable [50, 51, 70]. In other words, each eigenvector included in a spatial filtering model can be understood as an explanatory variable that is unrelated to other independent variables *and* accounts for the variation in the dependent variable (i.e., mortality).

Since the eigenvectors of ***MCM*** can be as many as *n* and all these eigenvectors are orthogonal to one another (i.e., mutually independent or no correlation), the next stage of spatial filtering is to select a parsimonious subset of eigenvectors. The conventional approaches to eigenvector selection are either to set a Moran’s I threshold for inclusion [50] or to apply stepwise regression to positive Moran’s I values [71]. However, both of these approaches used an iterative process, and this can be computationally demanding. To address the computational demands, Tiefelsdorf and Griffith [51] proposed to minimize a Z-score objective function of Moran’s I of residuals and they found that this new approach greatly facilitates the process of eigenvector selection and removes spatial autocorrelation. Furthermore, this approach guarantees that each eigenvector included in the regression is associated with the dependent variable. In this study, we use the Tiefelsdorf and Griffith [51] approach; details of how to implement this approach can be found in Chun and Griffith [72]. The spatial weight matrix was constructed based on the first order Queen specification (i.e., neighbors are defined as those counties that share the same boundary or a vertex).

The analytic strategy has several steps. First we examined descriptive statistics and Moran’s I tests for measuring spatial autocorrelation. Second, we estimated both OLS and spatial filtering regression models. The second step will help us to better understand whether individual dimensions of segregation are associated with mortality. In the third step, we focused on each dimension of segregation by including the same segregation dimension measures for three race/ethnic groups in one regression model, e.g., simultaneously considering white/black, white/Hispanic, and white/API entropy indices. The models were estimated with both OLS and spatial filtering approaches. Following Tiefelsdorf and Griffith [51], we only consider the eigenvectors that are statistically significant and help to explain the spatial variation in mortality. We used R for all statistical analyses in this study [73].

## Results

The descriptive statistics and Moran’s I values are shown in Table 1. The average age-sex standardized mortality rate in US counties was 8.9 deaths per 1,000 population between 2004 and 2008, which is comparable with the number in a recent report [74]. The Moran’s I of mortality was 0.55, suggesting that counties with similar mortality rates tend to cluster together and the strength of spatial autocorrelation is moderate. As for the segregation measures, two findings are notable. First, regardless of dimensions, white/black segregation measures are, on average, higher than white/Hispanic and white/API segregation indices. Second, all five dimensions of segregation were found to be spatially autocorrelated. Based on Moran’s I, the exposure and centralization dimensions have the strongest and weakest levels of global autocorrelation, respectively. This pattern is consistent across the three race/ethnic groups. We also computed the Pearson’s correlation coefficients between mortality and all fifteen segregation measures in (Table A in S1 File).

**Table 1:** Descriptive Statistics of the Variables of This Study.

	Min.	Max.	Mean	S.D.	Moran’s I^§^
Dependent Variable					
** Mortality (per 1,000 population)**	2.904	18.889	8.913	1.453	0.554
Independent Variables					
** Segregation**					
***Non-Hispanic White vs*. *Non-Hispanic Black***					
** **Entropy (evenness)	0.000	0.691	0.096	0.105	0.320
** **Isolation Index (exposure)	0.000	0.890	0.152	0.200	0.665
** **Absolute Centralization (centralization)	-0.763	0.955	0.271	0.299	0.099
** **Delta (concentration)	0.000	0.990	0.518	0.257	0.202
** **Spatial Proximity (clustering)	1.000	1.749	1.049	0.081	0.313
***Non-Hispanic White vs*. *Hispanic***					
** **Entropy (evenness)	0.000	0.442	0.054	0.068	0.306
** **Isolation Index (exposure)	0.001	0.971	0.128	0.169	0.662
** **Absolute Centralization (centralization)	-0.585	0.958	0.249	0.271	0.102
** **Delta (concentration)	0.000	0.945	0.460	0.235	0.159
** **Spatial Proximity (clustering)	1.000	1.911	1.030	0.058	0.324
***Non-Hispanic White vs*. *Asians/Pacific Islanders***					
** **Entropy (evenness)	0.000	0.426	0.043	0.043	0.230
** **Isolation Index (exposure)	0.000	0.632	0.028	0.058	0.446
** **Absolute Centralization (centralization)	-0.561	0.970	0.266	0.285	0.088
** **Delta (concentration)	0.000	0.986	0.496	0.242	0.132
** **Spatial Proximity (clustering)	1.000	1.368	1.008	0.022	0.350
** Metropolitan**					
** **Metropolitan Status (1 = yes, 0 = no)	0.000	1.000	0.349	0.477	N.A.
** Socioeconomic Status**					
** **Social Affluence	-7.676	5.382	0.000	1.000	0.479
** **Social Disadvantage	-2.842	10.166	0.000	1.000	0.362
** Racial Composition**					
** **% Non-Hispanic Black	0.000	0.868	0.089	0.144	0.802
** **% Hispanic	0.000	0.986	0.076	0.128	0.769
** **% other races	0.000	0.910	0.040	0.069	0.414
** Income Inequality**					
** **Gini	0.272	0.621	0.431	0.037	0.309
** Social Capital**					
** **Social Capital Index	-3.804	15.222	-0.002	1.642	0.580
** Population Health**					
** **Unhealthy Days per Month	0.000	16.500	6.822	2.573	0.391
** **Adult Obesity Rate	11.700	43.700	28.930	3.700	0.653

^§^All Moran’s I values are statistically significant at 0.01 level.

With respect to other covariates, roughly 35 percent of counties were metropolitan counties and overall (and on average), a county had approximately 9 percent of non-Hispanic blacks, 8 percent of Hispanics, and 4 percent of other minorities. The average Gini coefficient in our data, 0.43, closely matched the national level of income inequality [75]. Every month, the population in a county reported approximately 7 unhealthy days and the average adult obesity rate was almost 30 percent. All of the independent variables have moderate to strong spatial autocorrelations based on a Queen first order spatial weights matrix (see Moran’s I in Table 1). The spatial structure evident in our dependent and independent variables implies the need for model specifications that are explicitly spatial.

**Fig 1:**
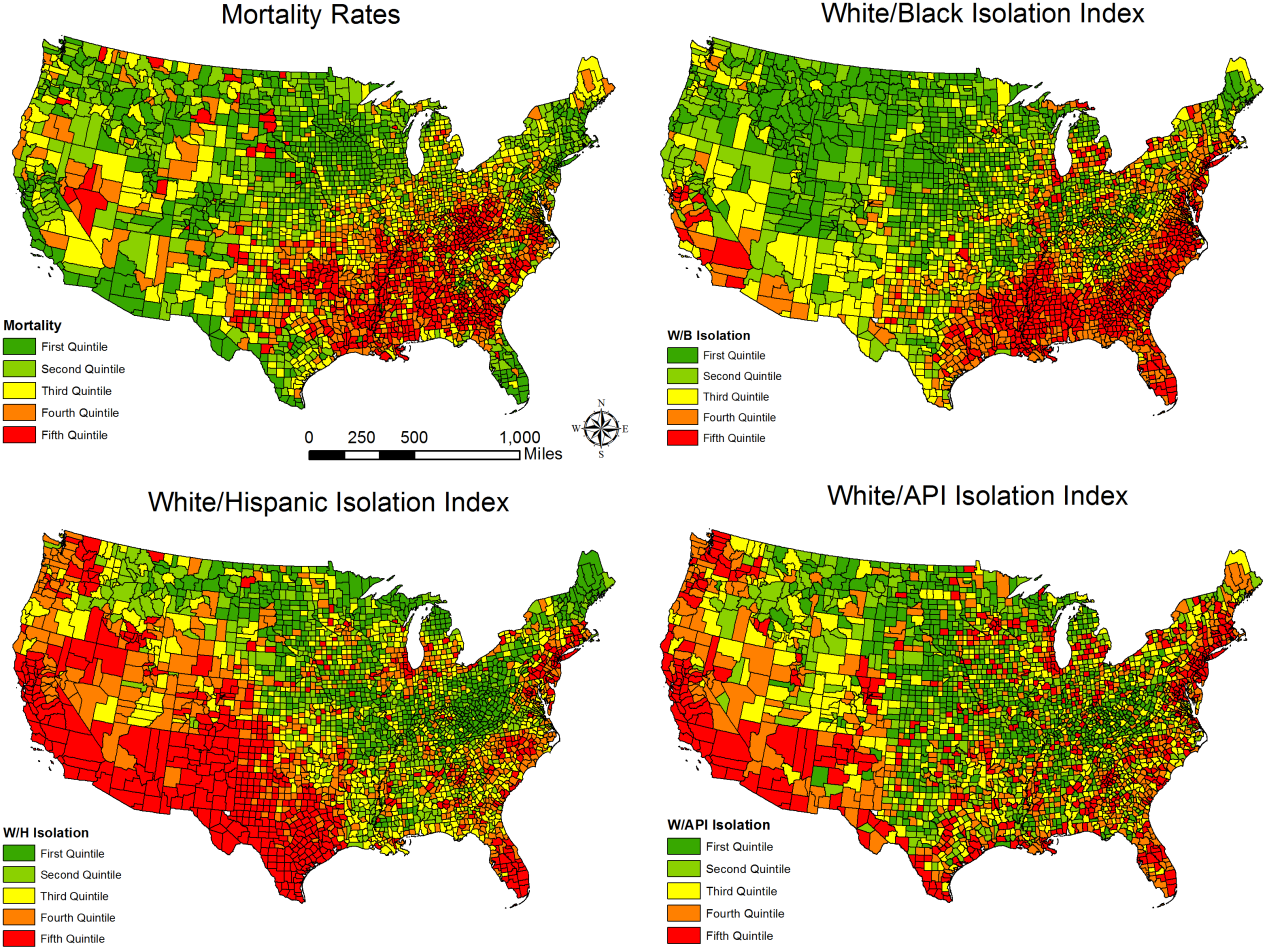
Spatial Distributions of mortality and three isolation indices (maps are created by the authors and the shapefiles are publicly available online).

Table 2 presents the OLS and spatial filtering regression results by race/ethnicity groups. There are several important findings. First, among the white/black segregation measures, only isolation index (the exposure dimension) was found to be positively related to mortality. While this association holds in both OLS and spatial filtering models, the estimated association between isolation and mortality decreased by more than 30 percent between the OLS and the spatial filtering model ((1.051–0.709)/1.051 = 0.33). Second, as we argued and expected, the white/Hispanic and white/API segregation measures are negatively associated with mortality; though the evidence for this is based only on the isolation index. Two segregation measures, white/Hispanic spatial proximity index and white/API absolute centralization, follow our expectations in the OLS models, but in the spatial filtering models there are no longer significant (see spatial filtering models in Table 2). That said, their associations with mortality in the OLS models are confounded with the eigenvectors (i.e., omitted variables) as taking the eigenvectors identified by the spatial filtering approach into account fully explained the association. Third, we visualized the spatial distributions of the three significant isolation indices and mortality rates by quintiles in Fig 1. The white/black isolation index and mortality rates share a similar pattern where the Black Belt, Mississippi Delta, and eastern Texas have both high mortality rates and white/black isolation. By contrast, white/Hispanic and white/API isolation indices are higher along with the US/Mexico border and Pacific coast, in which mortality rates are relatively low. Fig 1, to some extent, provides an explanation for why the associations of the exposure dimension of segregation with mortality differ by race/ethnic groups. Even considering other potential explanatory variables in the models, our results offered support to the bivariate visual comparison in Fig 1.

**Table 2:** OLS and Spatial Filtering Results by Race/ethnicity Dyads.

	Non-Hispanic Whites vs. Non-Hispanic Blacks				Non-Hispanic Whites vs. Hispanics				Non-Hispanic Whites vs. Asians/Pacific Islanders			
	OLS		Spatial Filtering		OLS		Spatial Filtering		OLS		Spatial Filtering	
	β	S.E.	β	S.E.	β	S.E.	β	S.E.	β	S.E.	β	S.E.
**Intercept**	5.827	0.571***	7.506	0.524***	6.575	0.669***	6.975	0.614***	4.320	1.713*	5.976	1.531***
**Segregation**												
Entropy (evenness)	-0.462	0.357	-0.158	0.327	0.293	0.509	0.249	0.493	-0.027	0.760	-0.278	0.699
Isolation Index (exposure)	1.051	0.173***	0.709	0.171***	-1.193	0.166***	-1.406	0.182***	-2.532	0.717***	-1.369	0.653*
Absolute Centralization (centralization)	-0.118	0.079	-0.034	0.070	-0.107	0.091	-0.071	0.081	-0.170	0.085*	-0.091	0.076
Delta (concentration)	-0.066	0.103	0.161	0.093	-0.088	0.110	0.163	0.100	-0.063	0.111	0.143	0.100
Spatial Proximity (clustering)	-0.267	0.466	-0.400	0.413	-1.135	0.578*	-0.044	0.525	1.265	1.687	1.023	1.506
**Metropolitan**												
Metropolitan Status	0.111	0.048*	0.095	0.043*	0.153	0.047**	0.113	0.043**	0.148	0.047**	0.112	0.042**
**Socioeconomic Status**												
Social Affluence	-0.485	0.027***	-0.501	0.025***	-0.443	0.027***	-0.460	0.025***	-0.406	0.028***	-0.440	0.026***
Social Disadvantage	0.330	0.028***	0.362	0.025***	0.291	0.028***	0.326	0.026***	0.351	0.023***	0.435	0.022***
**Racial Composition**												
% Non-Hispanic Black					1.652	0.175***	1.377	0.197***	1.306	0.161***	0.524	0.167**
% Hispanic	-2.097	0.164***	-2.684	0.177***					-1.704	0.173***	-2.580	0.189***
% Other Races	0.401	0.324	1.667	0.330***	1.104	0.328***	2.352	0.339***				
**Income Inequality**												
Gini	2.672	0.562***	-0.081	0.531	2.637	0.558***	-0.062	0.535	2.768	0.558***	0.167	0.534
**Social Capital**												
Social Capital Index	-0.158	0.014***	-0.016	0.014	-0.151	0.014***	-0.005	0.014	-0.166	0.014***	-0.033	0.014*
**Population Health**												
Unhealthy Days/Month	0.067	0.008***	0.051	0.008***	0.079	0.008***	0.055	0.008***	0.072	0.008***	0.051	0.008***
Adult Obesity Rates	0.063	0.007***	0.050	0.007***	0.062	0.007***	0.052	0.007***	0.060	0.007***	0.055	0.007***
**Eigenvector 15**	N.A.		7.010	0.873***	N.A.		6.995	0.876***	N.A.		6.857	0.870***
**Eigenvector 19**	N.A.		10.505	0.890***	N.A.		10.688	0.893***	N.A.		10.647	0.892***
**Eigenvector 1**	N.A.		-11.752	0.920***	N.A.		-10.808	0.932***	N.A.		-11.419	0.927***
**Eigenvector 6**	N.A.		11.124	0.981***	N.A.		11.023	0.992***	N.A.		10.478	0.984***
**Total Eigenvectors** ^‡^		N.A.		55		N.A.		58		N.A.		52
**Adjusted R-square**		0.551		0.657		0.552		0.656		0.557		0.657
**Residuals’ Moran’s I**		0.243***		0.038***		0.238***		0.037***		0.237***		0.036***

*p<0.05;

**p<0.01;

***p<0.001;

^**‡**^ **All eigenvectors are statistically significant (p<0.05).**

**N.A.: Not Applicable.**

**Fig 2:**
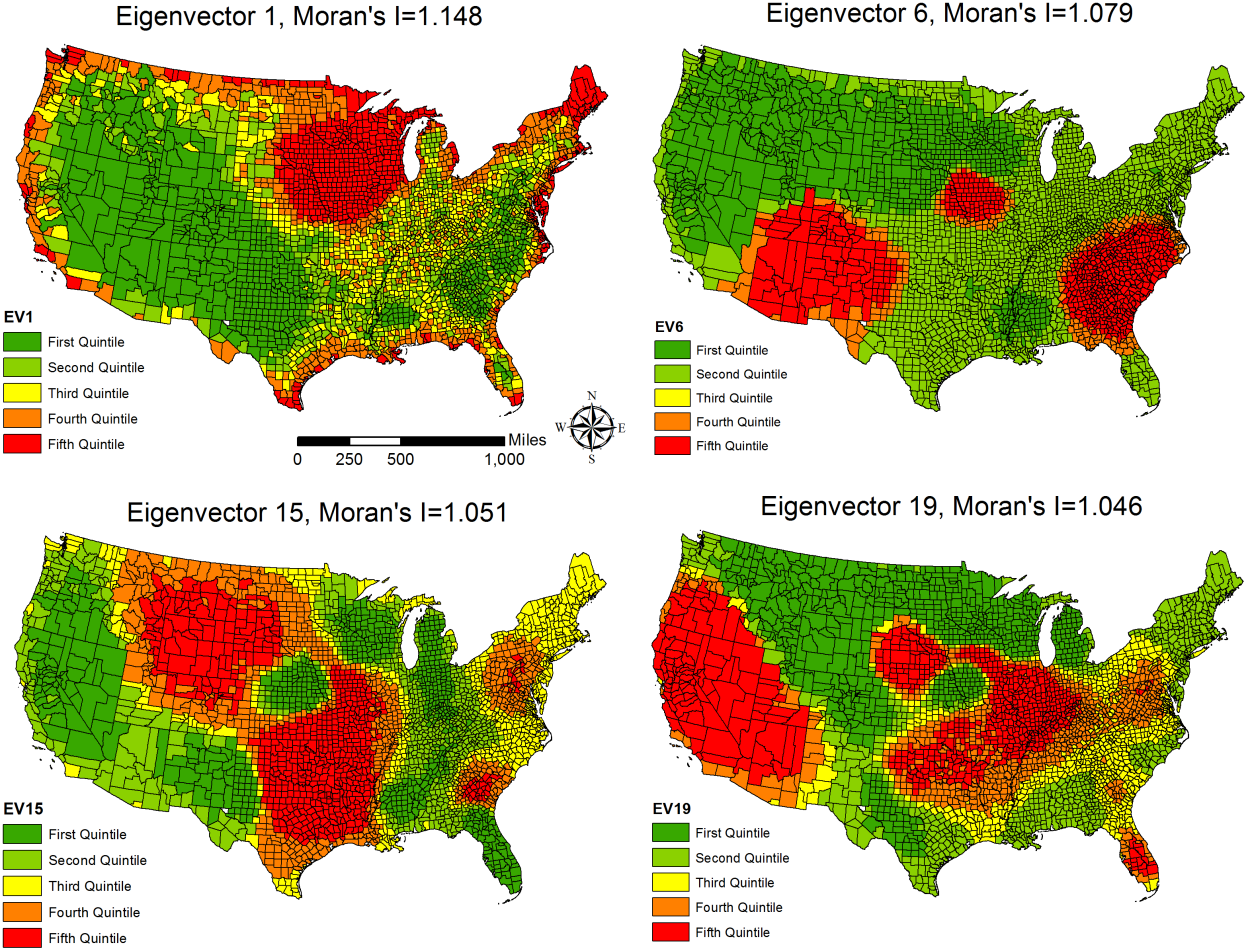
Spatial Patterns of the First Four Common Eigenvectors and their Moran’s I Values (maps are created by the authors and the shapefiles are publicly available online).

The spatial filtering approach identified more than 50 eigenvectors and improved the adjusted R-square by approximately 20 percent from the OLS models for each race/ethnicity group. After examining these eigenvectors (results not shown but available upon request), a fourth observation from our analysis is that the first four most important eigenvectors were the same across race/ethnic groups and they are eigenvectors 1, 6, 15, and 19. Following Griffith [49], we visualized these shared eigenvectors (based on quintiles) to gain a better understanding of what their spatial patterns are. As shown in Fig 2, the four eigenvectors have distinctive patterns. For example, eigenvector 15 suggests that the highest component values of this particular spatial pattern largely correspond to an area west of the Mississippi from Texas in the south through to the northern Plains and Mountain West (with the exception of parts of Nebraska). The Mid-Atlantic areas also have high values. The lowest components values are found in the west coast and an area covering parts of the Rust Belt, Appalachia, and Florida. The spatial pattern of eigenvector 15 seems to highlight the importance of Native American population and territories. Eigenvector 1 shows a pattern where the high component values surround all US borders and concentrate on Michigan, Wisconsin, Minnesota and Iowa. Eigenvector 1 seems to capture the combination of the edge effect and the spatial distribution of Mainline Protestants in the US (demonstrated in Fig A in S2 File). As discussed in the method section, the four eigenvectors are independent of one another and each represents a variable at the county level not considered in the analysis. Note that in the spatial filtering models, these eigenvectors were already included in the analysis and could be regarded as *additional* independent variables that represent the covariates omitted in regression but associated with mortality (e.g., migration trends). Their estimated relationships with mortality were in Table 2. Except eigenvector 1, all other eigenvectors are positively associated with mortality and the parameter estimates are stable across models. The estimates for these eigenvectors are relatively large due to the fact the eigenvalues in each eigenvectors are small (i.e., decomposition of errors).

Fifth, beyond the findings related to segregation, the associations of other independent variables with mortality in Table 2 echo recent findings in the mortality literature [8, 76]. For instance, both OLS and spatial filtering results indicated that metropolitan counties have higher mortality rates than nonmetropolitan counterparts, the so-called rural paradox [9]. Socioeconomic status variables suggested that better socioeconomic environment is associated with lower mortality, as Link and Phelan [29] argued. Regarding racial composition, the proportion of Hispanic population was negatively related to mortality, whereas the presence of other minority groups increased mortality. It should be noted that these associations between race/ethnicity groups and mortality were found even after taking racial segregation into account.

Finally, the statistically significant associations for both income inequality and social capital index with mortality observed in the OLS models were eliminated in the spatial filtering models. That is, once spatial structure is taking into account, the relevance of social capital and income inequality to mortality is reduced; as such, our findings contribute to the ongoing debates on these topics [60, 77].

Following our analytic strategy, we also implemented analyses by segregation dimensions and the results were summarized in Tables 3 and 4. Since the main interest of this study is segregation (and the findings related to other independent variables, such as metropolitan status and income inequality, were similar to those in Table 2), we focus our discussions on segregation and spatial filtering results. Again, there are several notable findings. First and foremost, the estimated relationships between the white/black segregation measures and mortality were all positive and they were statistically significant in four of the five segregation dimensions (except clustering). By contrast, the associations of white/API segregation measures with mortality were negative, with a statistically significant association for evenness, exposure, and clustering dimensions. While the white/Hispanic entropy index was marginally significant and negatively related to mortality, overall, white/Hispanic segregation does not affect all-cause mortality (in models including other covariates). Second, the OLS models seemed to overestimate the importance of segregation, such as the findings in the evenness and clustering dimensions. The spatial structure underlying the data contributes to this overestimation as the spatial filtering models generated weaker relationships between segregation measures and mortality, and improved the adjusted R-squared.

**Table 3:** OLS and Spatial Filtering Results by Segregation Dimensions (Evenness, Exposure, and Centralization).

	Evenness (entropy)				Exposure (isolation index)				Centralization (absolute centralization index)			
	OLS		Spatial Filtering		OLS		Spatial Filtering		OLS		Spatial Filtering	
	β	S.E.	β	S.E.	β	S.E.	β	S.E.	β	S.E.	β	S.E.
**Intercept**	5.454	0.327***	7.076	0.321***	5.552	0.326***	7.112	0.325***	5.533	0.326***	7.157	0.321***
**Segregation**												
Non-Hispanic Black/White	0.841	0.226***	0.624	0.208**	0.592	0.257*	0.486	0.238*	0.132	0.134	0.238	0.119*
Hispanic/White	-1.759	0.359***	-0.577	0.331†	-0.144	0.352	0.286	0.321	-0.126	0.167	-0.127	0.148
Asians/White	-1.826	0.520***	-1.098	0.468*	-2.687	0.433***	-2.275	0.406***	-0.220	0.144	-0.115	0.127
**Metropolitan**												
Metropolitan Status	0.127	0.047**	0.110	0.043**	0.128	0.048**	0.111	0.043**	0.132	0.047**	0.114	0.042**
**Socioeconomic Status**												
Social Affluence	-0.453	0.027***	-0.474	0.025***	-0.441	0.027***	-0.451	0.025***	-0.474	0.027***	-0.490	0.025***
Social Disadvantage	0.302	0.028***	0.354	0.026***	0.296	0.028***	0.333	0.026***	0.317	0.028***	0.359	0.026***
**Racial Composition**												
% Non-Hispanic Black	1.477	0.174***	0.790	0.179***	0.894	0.331**	0.626	0.337†	1.299	0.173***	0.771	0.177***
% Hispanics	-1.648	0.173***	-2.397	0.190***	-1.522	0.437***	-2.298	0.426***	-1.941	0.167***	-2.576	0.179***
% Other Races	0.939	0.329**	1.880	0.336***	1.122	0.343**	2.225	0.353***	0.581	0.329†	1.751	0.336***
**Income Inequality**												
Gini	2.746	0.559***	0.026	0.533	2.697	0.558***	0.082	0.534	2.694	0.556***	-0.031	0.529
**Social Capital**												
Social Capital Index	-0.156	0.014***	-0.020	0.014	-0.158	0.014***	-0.017	0.014	-0.158	0.014***	-0.019	0.014
**Population Health**												
Unhealthy Days/Month	0.071	0.008***	0.055	0.007***	0.069	0.008***	0.054	0.008***	0.069	0.008***	0.055	0.007***
Adult Obesity Rates	0.062	0.007***	0.051	0.007***	0.058	0.007***	0.046	0.007***	0.061	0.007***	0.049	0.007***
**Eigenvector 15**	N.A.		7.003	0.870***	N.A.		7.307	0.873***	N.A.		6.795	0.869***
**Eigenvector 19**	N.A.		10.409	0.886***	N.A.		10.662	0.887***	N.A.		10.490	0.887***
**Eigenvector 1**	N.A.		-11.039	0.923***	N.A.		-10.658	0.918***	N.A.		-11.457	0.913***
**Eigenvector 6**	N.A.		11.130	0.977***	N.A.		10.541	0.985***	N.A.		10.959	0.979***
**Eigenvector 21**	N.A.		6.883	0.887***	N.A.		6.942	0.882***	N.A.		6.901	0.876***
**Eigenvector 17**	N.A.		6.801	0.869***	N.A.		6.615	0.999***	N.A.		6.936	0.868***
**Total Eigenvectors** ^**‡**^		N.A.		53		N.A.		54		N.A.		54
**Adjusted R-square**		0.558		0.659		0.558		0.662		0.553		0.658
**Residuals’ Moran’s I**		0.237***		0.036***		0.244***		0.033***		0.243***		0.038***

^**†**^ **p<0.1;**

*p<0.05;

**p<0.01;

***p<0.001;

^**‡**^ **All eigenvectors are statistically significant (p<0.05).**

**N.A.: Not Applicable.**

**Table 4:** OLS and Spatial Filtering Results by Segregation Dimensions (Concentration and Clustering).

	Concentration (Delta index)				Clustering (spatial proximity index)			
	OLS		Spatial Filtering		OLS		Spatial Filtering	
	β	S.E.	β	S.E.	β	S.E.	β	S.E.
**Intercept**	5.621	0.327***	7.123	0.320***	10.282	0.971***	9.653	0.910***
**Segregation**								
Non-Hispanic Black/White	0.200	0.134	0.285	0.120*	0.589	0.278*	0.344	0.256
Hispanic/White	-0.445	0.162**	-0.103	0.146	-1.716	0.378***	-0.429	0.346
Asians/White	-0.003	0.149	-0.066	0.133	-3.632	0.959***	-2.576	0.878**
**Metropolitan**								
Metropolitan Status	0.125	0.047**	0.108	0.042*	0.138	0.047**	0.118	0.042**
**Socioeconomic Status**								
Social Affluence	-0.476	0.027***	-0.495	0.025***	-0.458	0.027***	-0.469	0.025***
Social Disadvantage	0.312	0.028***	0.353	0.026***	0.296	0.028***	0.351	0.026***
**Racial Composition**								
% Non-Hispanic Black	1.336	0.178***	0.858	0.180***	1.388	0.182***	0.885	0.200***
% Hispanics	-1.882	0.169***	-2.570	0.181***	-1.520	0.182***	-2.272	0.203***
% Other Races	0.663	0.330*	1.826	0.335***	0.987	0.330**	1.938	0.339***
**Income Inequality**								
Gini	2.574	0.557***	-0.081	0.528	2.703	0.556***	0.126	0.530
**Social Capital**								
Social Capital Index	-0.153	0.014***	-0.015	0.014	-0.157	0.014***	-0.019	0.014
**Population Health**								
Unhealthy Days	0.069	0.008***	0.053	0.008***	0.071	0.008***	0.057	0.007***
Adult Obesity Rates	0.061	0.007***	0.049	0.007***	0.059	0.007***	0.051	0.007***
**Eigenvector 15**	N.A.		6.861	0.867***	N.A.		6.804	0.871***
**Eigenvector 19**	N.A.		10.564	0.887***	N.A.		10.541	0.887***
**Eigenvector 1**	N.A.		-11.639	0.922***	N.A.		-10.968	0.921***
**Eigenvector 6**	N.A.		11.093	0.979***	N.A.		10.772	0.985***
**Eigenvector 21**	N.A.		7.167	0.883***	N.A.		6.693	0.880***
**Eigenvector 17**	N.A.		7.105	0.869***	N.A.		6.754	0.869***
**Total Eigenvectors** ^†^		N.A.		55		N.A.		55
**Adjusted R-square**		0.553		0.659		0.558		0.660
**Residuals’ Moran’s I**		0.241***		0.037***		0.239***		0.033***

^**†**^ **p<0.1;**

*p<0.05;

**p<0.01;

***p<0.001;

^**‡**^ **All eigenvectors are statistically significant (p<0.05).**

**N.A.: Not Applicable.**

**Fig 3:**
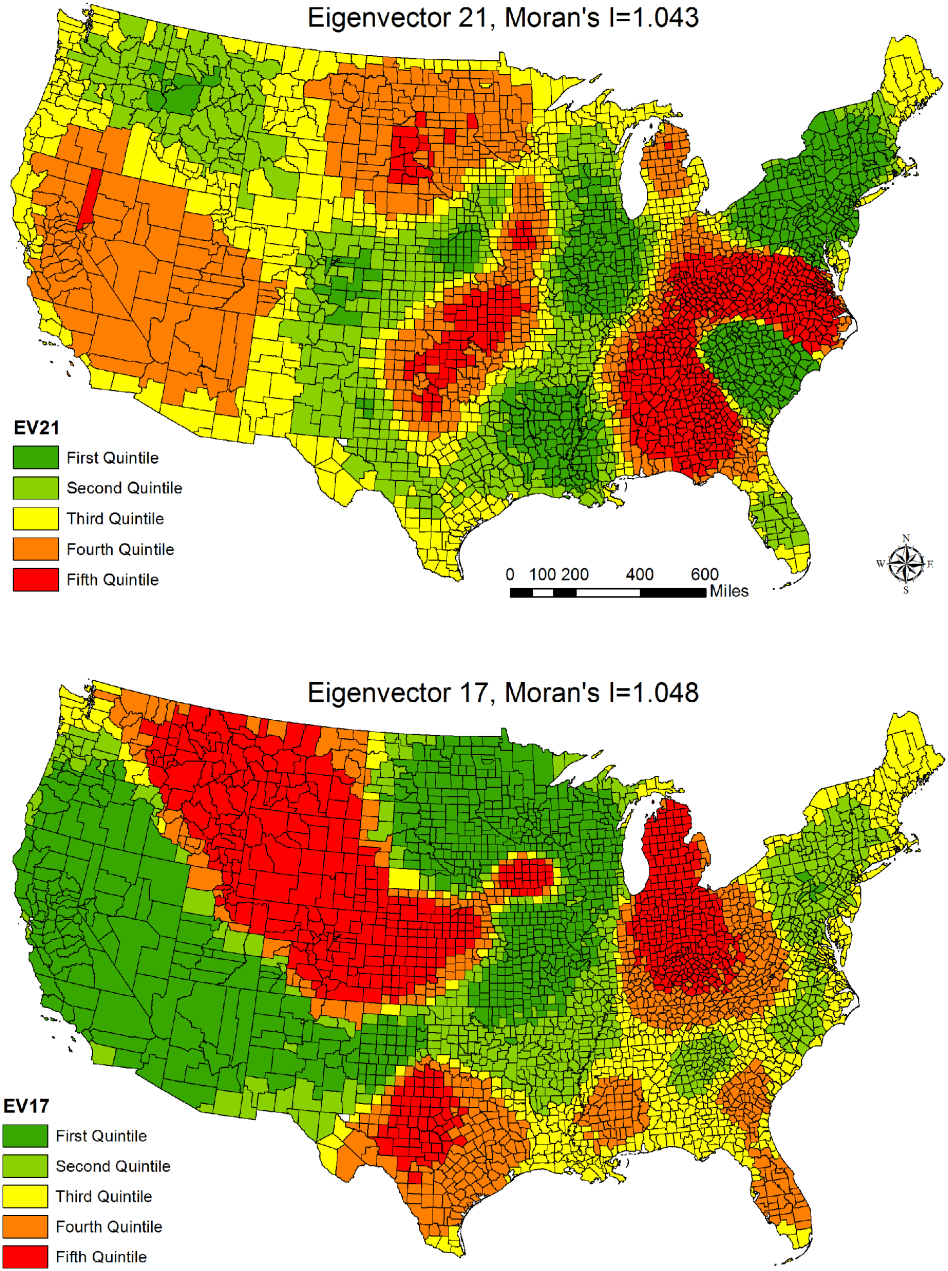
Spatial Patterns of the Two Additional Eigenvectors and Moran’s I Values (maps are created by the authors and the shapefiles are publicly available online).

Third, the total number of eigenvectors found in each model is comparable across segregation dimensions and, among them, six were commonly shared by the five segregation dimensions, i.e., eigenvectors 15, 19, 1, 6, 21, and 17. Comparing with the findings in Table 2, two additional eigenvectors, 21 and 17, were identified and they were shown in Fig 3. Again, both eigenvectors have spatial patterns that were different from those in Fig 2 and their associations with mortality were positive (see Tables 3 and 4). As seen in Figs 2 and 3, the Moran’s I values of the six most important eigenvectors were all positive, indicating that the high (low) component values of these eigenvectors are geographically close to one another. We would like to reiterate that the six eigenvectors capture the spatial processes that are not associated with the independent variables in the models but they contribute to the observed spatial pattern of mortality in the US.

Last, as the spatial filtering approach aims to remove spatial autocorrelation in the dependent variable, we conducted Moran’s I tests to assess if the residuals of these models are still spatially autocorrelated. The last row of Tables 2 and 3 indicated that spatial filtering effectively reduces spatial autocorrelation by approximately 85 percent from OLS models. The residuals’ Moran’s I values in spatial filtering models are all very close to zero (i.e., no spatial autocorrelation), while they remained statistically significant. The explanation for the statistical significance is that our eigenvector sets are optimal (based on statistical significance), rather than exhaustive [72]. When we included those eigenvectors with a p-value between 0.05 and 0.1, the residuals’ Moran’s I became non-significant (results not show but available). Since adding more eigenvectors does not change our findings, we believe the models presented in Tables 2 and 3 are the most parsimonious.

## Discussion and Conclusions

We used the findings above to examine the research hypotheses. Following the ethnic stratification perspective, we first hypothesized that white/black segregation is positively related to mortality. When taking all five segregation dimensions into account (Table 2), only the exposure dimension was found significantly and positively related to mortality. Nonetheless, the dimension-specific analyses (Tables 3 and 4) offered stronger evidence to support our hypothesis as four out of the five white/black segregation measures followed our expectation. In contrast to the literature [14], we have provided further evidence to suggest that most dimensions of white/black segregation would increase mortality even after adjusting for other covariates.

In addition, we also expected that the segregation between whites and non-black minority groups would be negatively related to mortality (i.e., beneficial) and that exposure dimension may be the most relevant determinant among the five dimensions. Our findings provided some support to our hypotheses. Specifically, the results in Table 2 suggested that higher levels of isolation between non-Hispanic whites and these two minority groups are associated with lower levels of mortality in US counties. Though white/Hispanic spatial proximity index (clustering dimension) and white/API absolute centralization index (centralization dimension) in the OLS models followed our expectations, these findings did not hold when the spatial structure underlying the data was considered. When considering one dimension of segregation at a time, we obtained stronger evidence for white/API than white/Hispanic segregation measures. Specifically, three of the five white/API dimensions of segregation were negatively related to mortality, whereas only the white/Hispanic entropy index was marginally significant. Overall, we received relatively weak support (in contrast to white/black segregation) for the argument that living in ethnic enclaves or communities may be beneficial for non-black minorities. Coupled with the recent findings by Dinwiddie and colleagues [33], we suggested that more efforts should be made to further understand whether the segregation between whites and non-black minorities can benefit population health.

The third hypothesis indicated that the spatial autocorrelation affects the estimates of the relationships between segregation and mortality and spatial filtering approach would identify the spatial patterns that are not only related to county-level mortality but also shared by various segregation dimensions. This hypothesis was confirmed as the OLS models tend to overestimate the importance of segregation (see both Tables 2 and 3). This pattern has been reported by several studies using a spatial perspective [8, 14, 48]. This study reinforces the suggestion that when analyzing ecological data a spatial analytic approach should be employed. Furthermore, the race/ethnicity-specific models shared four eigenvectors and the dimension-specific analyses identified two additional eigenvectors. The six eigenvectors have distinctive spatial patterns and each of them represents a dimension not captured by our proposed independent variables. While it is not clear what these covariates may be, they provide scientific insights into future mortality studies as researchers could explore what factors correspond to these spatial patterns [49]. For example, Eigenvector 1 in Fig 2 suggests the combination of the edge effect surrounding the US boundaries and the potential association of Mainline Protestants with mortality in the US (see the spatial patterns in Fig 2 and Fig A in S2 File). We would like to emphasize that these eigenvectors were already included in the spatial filtering models so it is not necessary to include new independent variables into the models, which is the advantage of applying the spatial filtering in empirical research [72].

Overall, we believe that our hypotheses received some support, especially from the spatial filtering models. This study includes several noteworthy findings. First, the race/ethnicity-specific analyses suggested that the relationship of isolation index with mortality is the most consistent among other segregation measures, which suggests that exposure to non-Hispanic whites may be the most important dimension of segregation. As Massey and Denton [11] defined, the exposure dimension refers to the extent to which minority and majority group members interact within a given area and the isolation index captures the level of segregation experienced by minorities. This definition fits the ethnic stratification and ethnic community/enclave perspectives and the opposite associations between non-Hispanic blacks and other minority groups were expected. Furthermore, the evenness and exposure dimensions of segregation were more closely related to mortality than the other three dimensions (Tables 3 and 4), which is consistent with Kramer and Hogue’s [13] emphasis on evenness and exposure dimensions of segregation in the literature on segregation and health.

Second, the relationships between white/Hispanic segregation and mortality were weakly supported by our analytic results. Though we suspected that white/Hispanic segregation measures may be highly correlated with racial composition or other independent variables, such as social capital and income inequality, our sensitivity analyses (not shown) where these variables were excluded did not support this explanation. Thus, the possible explanation would be that white/Hispanic segregation measures are associated with the spatial processes that are not included or captured by our models.

Our findings related to income inequality and social capital directly speak to but do not resolve some debates in ecological mortality research. Specifically, in Tables 2 and 3, we found that income inequality and social capital became non-significant determinants of mortality when using a spatial filtering approach, which contradicts several recent studies using other analytical techniques, such as spatial econometrics and weighted least squares modeling [7, 9, 55, 78]. One plausible explanation for the discrepancy is that inequality and social capital both have strong spatial patterns and when spatial filtering is used, these spatial patterns may be explained by the eigenvectors generated by the spatial filtering method. In other words, there may be some unknown covariates that confound the relationships between mortality and inequality and social capital. As the strength of spatial filtering is to identify potential covariates omitted in the analysis, our findings somewhat challenge the literature that income inequality and social capital matter for mortality. A systematic examination on why different spatial methods yield different conclusions on this topic is necessary. These intertwined substantive and methodological issues are beyond the scope of this current study but we recognize exploring this would be a fruitful future direction.

This study contributes to the mortality literature in the following ways. Only a few mortality studies have employed segregation as an explanation for mortality differentials across the US counties or cities [14, 24] and even less has focused on the segregation between whites and non-black minority groups, as well as the five dimensions of segregation. Using the ethnic stratification and ethnic community/enclave perspective, we argued that white/black segregation is detrimental to overall mortality but white/Hispanic and white/API segregation are beneficial. It should be emphasized that the empirical support (and findings) for our arguments was obtained even after controlling for county-level population health measures, which strongly validates our conclusions. In addition, the spatial filtering approach identified several common eigenvectors that demonstrate unique spatial patterns in US counties. These eigenvectors represent missing variables that have implications for mortality but cannot be captured with any of the independent variables in our models. That said, mortality researchers should think outside the box to find determinants of geographic mortality differentials and these unique patterns offer some clues.

We included several health infrastructure variables, such as numbers of medical doctors or hospital beds per 1,000 population, into the analysis to see if they account for the relationship between segregation and mortality. Similar to the findings reported by Kindig and Cheng [43], we did not find significant associations between county-level health infrastructure and mortality (not shown but available upon request). Our conclusions related to segregation and mortality are not altered, suggesting that our findings are robust and consistent. Moreover, the spatial dependence embedded in our data (both mortality and independent variables) is confirmed even using different types of spatial weight matrix, such as first order Rook or second order Queen. As there is no agreement on which spatial weight matrix should be used, this study follows previous literature [9, 14, 48] to report the findings based on the first order Queen approach.

There are several limitations specific to this study. First, the ecological relationships between segregation measures and mortality cannot be generalized to individuals, and we would urge health researchers to investigate how the health of non-black minorities is affected by segregation from non-Hispanic whites. Second, this study combined multiple data sources from different time points to explore the associations between the independent and dependent variables. No causality can be derived from our analyses and the temporal misalignment should be noted. Third, like other ecological studies, our analysis is subject to the modifiable area unit problem [79] as changing the unit of analysis (e.g., using tracts) may alter the findings and conclusions. Fourth, somewhat related to the previous limitation, this study used the NCHS county-level vital statistics to study the persistent US mortality spatial pattern. While some state governments offer vital statistics at the tract-level, these data do not allow a nationwide study. Future efforts are warranted to investigate the segregation-mortality association at other geographic levels.

Fifth, this study used all-cause age-sex standardized mortality as the dependent variable as it is an overall evaluation of population health in a county. While race-specific mortality rates can be calculated, we encountered the small area/population estimation issues [80] as numerous counties have zero death for non-black minorities. The NCHS also suggested that mortality rates with fewer than 20 age-specific deaths should be suppressed for statistical reliability, which further complicates the small area issue. This problem will also make it difficult to compute the racial/ethnic mortality difference within a county. A related issue is that we did not exclude those counties with relatively small minority residents (e.g., less than 1,000 minorities) and thus those counties with the more unstable mortality in our analysis. We note that some segregation measures such as the isolation index, our measure of exposure, may be more sensitive to the size of minority groups. While the approach we adopt is commonly used [14, 35], our findings may be subject to underestimated segregation indices [35]. A potential solution is to concentrate on one or two states with large Hispanic population, such as California or Arizona and examine the relationships between age-race-specific mortality rates and segregation (but the total number of counties would be low).

Finally, while our findings suggested that evenness and exposure dimensions are more important than others, future research may still need to investigate whether hypersegregation [81, 82] is a more useful measure in health research. Some segregation measures, such as absolute centralization index, may work better in metropolitan counties than nonmetropolitan counties, which may be one reason why we did not find significant findings for these dimensions (though we note that most nonmetropolitan counties include urban places, including county seats). As one of our goals is to understand whether the relationship between segregation and mortality varies by individual dimension of segregation, the analysis using hypersegregation is beyond the scope of our study. Similarly, several scholars have developed spatial segregation indices [36, 83, 84]; however, these measures have not been commonly used in mortality research. Questions as to whether the choice of segregation measures matters remain underexplored [85].

Some future research directions can be drawn from this study. First, our discussion on ethnic enclave and community is relevant to the literature on immigrant health [86, 87]. The Compressed Mortality Files used in this study do not include the information on nativity of the deceased, which prevents us from directly addressing this issue. Future studies should use other data sources to investigate the relationship among immigration, segregation, and health. Second, most mortality research used the latest available data to explain the mortality differentials across social groups and long-term latency between disease onset and death has been overlooked [88]. That is, the mortality differentials observed today may be the result of the socioeconomic or environmental factors in existence decades prior rather than those measured concurrently with mortality. Addressing the latency issue may better clarify the causality between the persistent mortality pattern and its determinants. Third, the segregation measures used in this study only concern two race/ethnic groups. The measures of multi-group segregation [89] and spatial segregation measures [36, 83] should be employed to understand whether the choice and specification of segregation measure alters the results/conclusions. Finally, more attention should be paid to the mortality ratios between different race/ethnicity groups (e.g., white and black) and their associations with two-group racial/ethnic segregation measures. The mortality ratios measure racial/ethnic health disparities within an area and using these ratios as dependent variables would speak directly to the important question of whether segregation is associated with racial/ethnic disparities. Moreover, examining the patterns and trends in race-specific mortality ratios and racial/ethnic health disparities overtime should be a research priority.

In sum, racial segregation is argued to be the major cause of health disparities [26] and a determinant of health outcomes [13]. Previous evidence has been drawn heavily from the non-Hispanic blacks. A growing body of literature has found that segregation may be beneficial to health outcomes or behaviors for non-black minorities, i.e., Hispanics and Asians/Pacific Islanders [90–92]. This study echoes the recent development in the literature and offers county-level evidence for the potential benefit of segregation for Hispanics and Asians/Pacific Islanders.

## Supporting Information

S1 File Pearson’s Correlation Coefficients between Mortality and Segregation Indices (Table A). (DOCX)

S2 File Spatial Distribution of Mainline Protestants in US counties, by Quintiles (Fig A). (DOCX)

## References

[1] Hoyert DL. 75 Years of Mortality in the United States, 1935–2010. In: NCHS, editor. NCHS Data Brief2012. 22617094

[2] Hoyert DL, Xu J. Deaths: preliminary data for 2011. Natl Vital Stat Rep. 2012;61(6):1–52. 24984457

[3] Cossman JS, Cossman RE, James WL, Campbell CR, Blanchard TC, Cosby AG. Persistent clusters of mortality in the United States. American journal of public health. 2007;97(12):2148 17538052 10.2105/AJPH.2006.093112 PMC2089111

[4] Blanchard TC, Bartkowski JP, Matthews TL, Kerley KR. Faith, morality and mortality: The ecological impact of religion on population health. Social Forces. 2008;86(4):1591–620.

[5] Cossman RE, Cossman JS, Cosby AG, Reavis RM. Reconsidering the rural–urban continuum in rural health research: a test of stable relationships using mortality as a health measure. Population Research and Policy Review. 2008;27(4):459–76.

[6] Lochner KA, Kawachi I, Brennan RT, Buka SL. Social capital and neighborhood mortality rates in Chicago. Social science & medicine. 2003;56(8):1797–805. 12639596 10.1016/s0277-9536(02)00177-6

[7] McLaughlin DK, Stokes CS, Smith PJ, Nonoyama A. Differential mortality across the U.S.: The influence of place-based inequality In: Linda M. Lobao, Gregory Hooks, Tickamyer AR, editors. The sociology of spatial inequality. Albany, NY: SUNY Press; 2007 p. 141–62.

[8] Sparks PJ, Sparks CS. An application of spatially autoregressive models to the study of US county mortality rates. Population, Space and Place. 2010;16(6):465–81. 764429494; 201066936.

[9] Yang TC, Jensen L, Haran M. Social capital and human mortality: Explaining the rural paradox with county-level mortality data. Rural Sociology. 2011;76(3):347–74. 25392565 10.1111/j.1549-0831.2011.00055.x PMC4225697

[10] White K, Borrell LN. Racial/ethnic residential segregation: framing the context of health risk and health disparities. Health & place. 2011;17(2):438–48. 21236721 10.1016/j.healthplace.2010.12.002 PMC3056936

[11] Massey DS, Denton NA. The dimensions of residential segregation. Social forces. 1988;67(2):281–315.

[12] Acevedo-Garcia D, Lochner KA, Osypuk TL, Subramanian SV. Future directions in residential segregation and health research: a multilevel approach. American Journal of Public Health. 2003;93(2):215–21. 12554572 10.2105/ajph.93.2.215 PMC1447719

[13] Kramer MR, Hogue CR. Is segregation bad for your health? Epidemiologic reviews. 2009;31(1):178–94. 19465747 10.1093/epirev/mxp001 PMC4362512

[14] Sparks JP, Sparks CS, Campbell JJA. An application of Bayesian spatial statistical methods to the study of racial and poverty segregation and infant mortality rates in the US. Geojournal. 2013;78:389–405.

[15] Fischer MJ. The relative importance of income and race in determining residential outcomes in US urban areas, 1970–2000. Urban Affairs Review. 2003;38(5):669–96.

[16] Logan JR, Stults BJ, Farley R. Segregation of minorities in the metropolis: Two decades of change. Demography. 2004;41(1):1–22. 15074122 10.1353/dem.2004.0007

[17] Wilkes R, Iceland J. Hypersegregation in the twenty-first century. Demography. 2004;41(1):23–36. 15074123 10.1353/dem.2004.0009

[18] Murdock SH, Hwang SS, Hoque M. Nonmetropolitan residential segregation revisited. Rural sociology. 1994;59(2):236–54.

[19] Fuguitt GV. Population change in nonmetropolitan America: Center for Demography and Ecology, University of Wisconsin—Madison; 1994.

[20] Parisi D, Lichter DT, Taquino MC. Multi-scale residential segregation: Black exceptionalism and America's changing color line. Social Forces. 2011;89(3):829–52.

[21] Johnson KM, Lichter DT. Growing diversity among America's children and youth: Spatial and temporal dimensions. Population and Development Review. 2010;36(1):151–76.

[22] Lobao LM, Hooks G, Tickamyer AR. The Sociology of Spatial Inequality: State University of New York Press; 2007.

[23] Cressie NAC. Statistics for Spatial Data. London, UK: John Wiley & Sons, Inc; 1991.

[24] Collins CA, Williams DR, editors. Segregation and mortality: The deadly effects of racism? Sociological Forum; 1999: Springer.

[25] Logan JR. Growth, politics, and the stratification of places. American Journal of Sociology. 1978:404–16.

[26] Williams DR, Collins C. Racial residential segregation: a fundamental cause of racial disparities in health. Public health reports. 2001;116(5):404 12042604 10.1093/phr/116.5.404 PMC1497358

[27] Brulle RJ, Pellow DN. Environmental justice: human health and environmental inequalities. Annu Rev Public Health. 2006;27:103–24. 16533111 10.1146/annurev.publhealth.27.021405.102124

[28] Glaeser E, Vigdor J. The End of the Segregated Century: Racial Separation in America's Neighborhoods, 1890–2010: Manhattan Institute for Policy Research; 2012.

[29] Link BG, Phelan J. Social conditions as fundamental causes of disease. Journal of health and social behavior. 1995;35:80–94. 7560851

[30] Greenberg M, Schneider D. Violence in American cities: young black males in the answer, but what was the question? Social Science & Medicine. 1994;39(2):179–87. 8066496 10.1016/0277-9536(94)90326-3

[31] LeClere FB, Rogers RG, Peters KD. Ethnicity and mortality in the United States: individual and community correlates. Social Forces. 1997;76(1):169–98.

[32] Gaskin DJ, Dinwiddie GY, Chan KS, McCleary RR. Residential segregation and the availability of primary care physicians. Health services research. 2012;47(6):2353–76. 10.1111/j.1475-6773.2012.01417.x 22524264 PMC3416972

[33] Dinwiddie GY, Gaskin DJ, Chan KS, Norrington J, McCleary R. Residential segregation, geographic proximity and type of services used: evidence for racial/ethnic disparities in mental health. Social Science & Medicine. 2013;80:67–75. 23312305 10.1016/j.socscimed.2012.11.024 PMC4119020

[34] Hobbs F, Stoops N. Demographic trends in the 20th century: US Census Bureau; 2002.

[35] Iceland J, Weinberg DH, Steinmetz E. Racial and ethnic residential segregation in the United States 1980–2000: Bureau of Census; 2002.

[36] Lee BA, Reardon SF, Firebaugh G, Farrell CR, Matthews SA, O'Sullivan D. Beyond the Census Tract: Patterns and Determinants of Racial Segregation at Multiple Geographic Scales. American Sociological Review. 2008;73(5):766–91. 10.1177/000312240807300504 25324575 PMC4196718

[37] Logan JR, Zhang W, Alba RD. Immigrant enclaves and ethnic communities in New York and Los Angeles American sociological review. 2002:299–322.

[38] Song L, Son J, Lin N. Social Capital and Health In: Cockerham WC, editor. The New Blackwell Companion to Medical Sociology. Malden, MA: Blackwell; 2010 p. 184–210.

[39] Eschbach K, Ostir GV, Patel KV, Markides KS, Goodwin JS. Neighborhood context and mortality among older Mexican Americans: is there a barrio advantage? American journal of public health. 2004;94(10):1807–12. 15451754 10.2105/ajph.94.10.1807 PMC1448538

[40] Lee M-A, Ferraro KF. Neighborhood residential segregation and physical health among Hispanic Americans: Good, bad, or benign? Journal of health and social behavior. 2007;48(2):131–48. 17583270 10.1177/002214650704800203

[41] Bécares L, Nazroo J, Stafford M. The buffering effects of ethnic density on experienced racism and health. Health & place. 2009;15(3):700–8. 10.1016/j.healthplace.2008.10.008 19117792

[42] Whitley R, Prince M, McKenzie K, Stewart R. Exploring the ethnic density effect: a qualitative study of a London electoral ward. International journal of social psychiatry. 2006;52(4):376–91. 17262983 10.1177/0020764006067239

[43] Kindig DA, Cheng ER. Even as mortality fell in most US counties, female mortality nonetheless rose in 42.8 percent of counties from 1992 to 2006. Health Affairs. 2013;32(3):451–8. 10.1377/hlthaff.2011.0892 23459723

[44] Cressie NAC. Statisticsfor spatial data. John Willey&Sons 1993.

[45] Matthews SA, Parker DM. Progress in Spatial Demography. Demographic Research. 2013;28. 10.4054/demres.2013.28.10 PMC7454172 32863759

[46] Kawachi I, Subramanian SV, Kim D. Social Capital and Health. Springer New York; 2008 p. 1–26.

[47] Voss PR. Demography as a spatial social science. Population Research and Policy Review. 2007;26(5):457.

[48] Voss PR, Long DD, Hammer RB, Friedman S. County child poverty rates in the US: a spatial regression approach. Population Research and Policy Review. 2006;25(4):369–91.

[49] Griffith DA. Spatial autocorrelation and spatial filtering: gaining understanding through theory and scientific visualization: Springer; 2003.

[50] Griffith DA. Eigenfunction properties and approximations of selected incidence matrices employed in spatial analyses. Linear Algebra and its Applications. 2000;321(1):95–112.

[51] Tiefelsdorf M, Griffith DA. Semiparametric filtering of spatial autocorrelation: the eigenvector approach. Environment and Planning A. 2007;39(5):1193.

[52] Thayn JB, Simanis JM. Accounting for spatial autocorrelation in linear regression models using spatial filtering with eigenvectors. Annals of the Association of American Geographers. 2013;103(1):47–66.

[53] NCHS. Compressed Mortality File, 1999–2007 (machine readable data file and documentation, CD-ROM series 20, No.2M). Hyattsville, Maryland: National Center for Health Statistics; 2010.

[54] Yang TC, Chen YJ, Shoff C, Matthews SA. Using quantile regression to examine the effects of inequality across the mortality distribution in the US. Social science & medicine. 2012;74(12):1900–10. 22497847 10.1016/j.socscimed.2012.02.029 PMC3568764

[55] Yang TC, Teng HW, Haran M. The Impacts of Social Capital on Infant Mortality in the US: A Spatial Investigation. Applied spatial analysis and policy. 2009;2(3):211.

[56] Theil H. Statistical decomposition analysis: With applications in the social and administrative sciences: North-Holland Publishing Company Amsterdam; 1972.

[57] Duncan OD, Cuzzort RP, Duncan B. Statistical geography: Problems in analyzing areal data. Statistical geography: Problems in analyzing areal data. 1961.

[58] White MJ. Segregation and diversity measures in population distribution. Population index. 1986;52(2):198–221. 12340704

[59] Sampson RJ, Raudenbush SW, Earls F. Neighborhoods and violent crime: A multilevel study of collective efficacy. Science. 1997;277(5328):918–24. 9252316 10.1126/science.277.5328.918

[60] Lynch J, Smith GD, Harper S, Hillemeier M, Ross N, Kaplan GA, et al Is income inequality a determinant of population health? Part 1. A systematic review. Milbank Quarterly. 2004;82(1):5–99. 15016244 10.1111/j.0887-378X.2004.00302.x PMC2690209

[61] Sethi R, Somanathan R. Inequality and segregation. Journal of Political Economy. 2004;112(6):1296–321.

[62] Rupasingha A, Goetz SJ, Freshwater D. The production of social capital in US counties. Journal of Socio-Economics. 2006;35(1):83–101.

[63] Putnam RD. Bowling alone: The collapse and revival of American community: Simon and Schuster; 2001.

[64] Rupasingha A, Goetz SJ. US County-Level Social Capital Data, 1990–2005. University Park: The Northeast Regional Center for Rural Development, Penn State University; 2008 [cited 2011 08/17].

[65] County Health Rankings [Internet]. 2011 [cited January, 2013]. Available from: www.countyhealthrankings.org.

[66] CDC. Measuring Healthy Days: Population Assessment of health-related Quality of Life. In: Services DoHaH, editor. Atlanta, GA2000.

[67] CDC. Overweight and Obesity: Causes and Consequences 2012 [updated April 27, 2012; cited 2014 February 20]. Available from: http://www.cdc.gov/obesity/adult/defining.html.

[68] CDC. Behavioral Risk Factor Surveillance System 2013 [updated December 24, 2013; cited 2014 February 20]. Available from: http://www.cdc.gov/brfss/.

[69] Moran PAP. Notes on continuous stochastic phenomena. Biometrika. 1950;37(1/2):17–23. 15420245

[70] Getis A, Griffith DA. Comparative spatial filtering in regression analysis. Geographical analysis. 2002;34(2):130–40.

[71] Griffith DA, Peres-Neto PR. Spatial modeling in ecology: the flexibility of eigenfunction spatial analyses. Ecology. 2006;87(10):2603–13. 17089668 10.1890/0012-9658(2006)87[2603:smietf]2.0.co;2

[72] Chun Y, Griffith DA. Spatial Statistics and Geostatistics: Theory and Applications for Geographic Information Science and Technology: SAGE; 2013.

[73] The R Core Development Team. R: A language and environment for statistical computing. ISBN 3-900051-07-0. R Foundation for Statistical Computing Vienna, Austria, 2013 url: http://www.R-project.org; 2013.

[74] NCHS. Health, United States, 2011: With Special Feature on Socioeocnomic Status and Health. Hyattsville, MD: Library of Congress Catalog Number 76–641496; 2012.

[75] DeNavas-Walt C, Proctor B, Smith J. Income, poverty, and health insurance coverage in the United States: 2008 In: Bureau USC, editor. Washington, DC: U.S. Government Printing Office; 2009.

[76] Yang TC, Noah AJ, Shoff C. Exploring Geographic Variation in US Mortality Rates Using a Spatial Durbin Approach. Population, Space and Place. Forthcoming. 10.1002/psp.1809 PMC4310504 25642156

[77] Kawachi I, Kennedy BP, Lochner K, Prothrow-Stith D. Social capital, income inequality, and mortality. American journal of public health. 1997;87(9):1491–8. 9314802 10.2105/ajph.87.9.1491 PMC1380975

[78] McLaughlin DK, Stokes CS. Income inequality and mortality in US counties: does minority racial concentration matter? American journal of public health. 2002;92(1):99–104. 11772770 10.2105/ajph.92.1.99 PMC1447397

[79] Openshaw S. Ecological fallacies and the analysis of areal census data. Environment and Planning A. 1984;16(1):17–31. 12265900 10.1068/a160017

[80] Ghosh M, Rao J. Small area estimation: an appraisal. Statistical science. 1994:55–76.

[81] Massey DS, Denton NA. Hypersegregation in US metropolitan areas: Black and Hispanic segregation along five dimensions. Demography. 1989;26(3):373–91. 2792476

[82] Osypuk TL, Acevedo-Garcia D. Are racial disparities in preterm birth larger in hypersegregated areas? American Journal of Epidemiology. 2008;167(11):1295–304. 10.1093/aje/kwn043 18367470

[83] Reardon SF, Matthews SA, O’Sullivan D, Lee BA, Firebaugh G, Farrell CR, et al The geographic scale of metropolitan racial segregation. Demography. 2008;45(3):489–514. 18939658 10.1353/dem.0.0019 PMC2831394

[84] Kershaw KN, Osypuk TL, Do DP, De Chavez PJ, Roux AVD. Neighborhood-level racial/ethnic residential segregation and incident cardiovascular disease: the multi-ethnic study of atherosclerosis. Circulation. 2014:CIRCULATIONAHA. 114.011345. 10.1161/CIRCULATIONAHA.114.011345 PMC4293329 25447044

[85] Kramer MR, Cooper HL, Drews-Botsch CD, Waller LA, Hogue CR. Do measures matter? Comparing surface-density-derived and census-tract-derived measures of racial residential segregation. International journal of health geographics. 2010;9(1):29. 20540797 10.1186/1476-072X-9-29 PMC2898812

[86] Jasso G, Massey DS, Rosenzweig MR, Smith JP. Immigrant health: selectivity and acculturation. Critical perspectives on racial and ethnic differences in health in late life. 2004:227–66.

[87] Kandula NR, Kersey M, Lurie N. Assuring the health of immigrants: what the leading health indicators tell us. Annu Rev Public Health. 2004;25:357–76. 15015925 10.1146/annurev.publhealth.25.101802.123107

[88] Matthews SA. Epidemiology using a GIS: the need for caution. Computers, Environment and Urban Systems. 1990;14(3):213–21.

[89] Reardon SF, Firebaugh G. Measures of multigroup segregation. Sociological methodology. 2002;32(1):33–67.

[90] Yang TC, Shoff C, Noah AJ, Black N, Sparks CS. Racial segregation and maternal smoking during pregnancy: a multilevel analysis using the racial segregation interaction index. Social Science & Medicine. 2014;107:26–36. 24602968 10.1016/j.socscimed.2014.01.030 PMC4029363

[91] Osypuk TL, Diez Roux AV, Hadley C, Kandula NR. Are immigrant enclaves healthy places to live? The Multi-ethnic Study of Atherosclerosis. Social science & medicine. 2009;69(1):110–20. 19427731 10.1016/j.socscimed.2009.04.010 PMC2750873

[92] Walton E. Residential segregation and birth weight among racial and ethnic minorities in the United States. Journal of Health and Social Behavior. 2009;50(4):427–42. 20099449 10.1177/002214650905000404

