# Supplementary material for: Death by Segregation: Does the Dimension of Racial Segregation Matter?
Source: PLoS One. 2015 Sep 23;10(9):e0138489. doi: 10.1371/journal.pone.0138489 (PMC4580431; doi:10.1371/journal.pone.0138489)
Supplement: S1 File — (DOCX) [file pone.0138489.s001.docx]

Table A. Pearson’s Correlation Coefficients between Mortality and Segregation Indices

|  | Mortality | 1 | 2 | 3 | 4 | 5 | 6 | 7 | 8 | 9 | 10 | 11 | 12 | 13 | 14 | 15 |
| --- | --- | --- | --- | --- | --- | --- | --- | --- | --- | --- | --- | --- | --- | --- | --- | --- |
| Mortality | 1 |  |  |  |  |  |  |  |  |  |  |  |  |  |  |  |
| *Non-Hispanic Black* |  |  |  |  |  |  |  |  |  |  |  |  |  |  |  |  |
| 1. Entropy (evenness) | .084^**^ | 1 |  |  |  |  |  |  |  |  |  |  |  |  |  |  |
| 2. Isolation Index (exposure) | .354^**^ | .628^**^ | 1 |  |  |  |  |  |  |  |  |  |  |  |  |  |
| 3. Absolute Centralization  (centralization) | -.063^**^ | .338^**^ | .150^**^ | 1 |  |  |  |  |  |  |  |  |  |  |  |  |
| 4. Delta (concentration) | -.061^**^ | .479^**^ | .070^**^ | .632^**^ | 1 |  |  |  |  |  |  |  |  |  |  |  |
| 5. Spatial Proximity (clustering) | .154^**^ | .828^**^ | .754^**^ | .234^**^ | .252^**^ | 1 |  |  |  |  |  |  |  |  |  |  |
| *Hispanic* |  |  |  |  |  |  |  |  |  |  |  |  |  |  |  |  |
| 6. Entropy (evenness) | -.082^**^ | .591^**^ | .417^**^ | .315^**^ | .339^**^ | .501^**^ | 1 |  |  |  |  |  |  |  |  |  |
| 7. Isolation Index (exposure) | -.145^**^ | .278^**^ | .218^**^ | .205^**^ | .171^**^ | .272^**^ | .599^**^ | 1 |  |  |  |  |  |  |  |  |
| 8. Absolute Centralization  (centralization) | -.083^**^ | .337^**^ | .153^**^ | .886^**^ | .604^**^ | .250^**^ | .348^**^ | .243^**^ | 1 |  |  |  |  |  |  |  |
| 9. Delta (concentration) | -.112^**^ | .352^**^ | .084^**^ | .620^**^ | .830^**^ | .211^**^ | .488^**^ | .283^**^ | .669^**^ | 1 |  |  |  |  |  |  |
| 10. Spatial Proximity (clustering) | -.128^**^ | .394^**^ | .273^**^ | .267^**^ | .269^**^ | .364^**^ | .815^**^ | .681^**^ | .292^**^ | .383^**^ | 1 |  |  |  |  |  |
| *Asians/Pacific Islanders* |  |  |  |  |  |  |  |  |  |  |  |  |  |  |  |  |
| 11. Entropy (evenness) | -.008 | .513^**^ | .399^**^ | .371^**^ | .397^**^ | .451^**^ | .524^**^ | .331^**^ | .378^**^ | .410^**^ | .394^**^ | 1 |  |  |  |  |
| 12. Isolation Index (exposure) | -.184^**^ | .435^**^ | .349^**^ | .269^**^ | .228^**^ | .408^**^ | .543^**^ | .503^**^ | .301^**^ | .278^**^ | .492^**^ | .731^**^ | 1 |  |  |  |
| 13. Absolute Centralization  (centralization) | -.071^**^ | .313^**^ | .156^**^ | .860^**^ | .595^**^ | .223^**^ | .310^**^ | .212^**^ | .891^**^ | .626^**^ | .262^**^ | .390^**^ | .284^**^ | 1 |  |  |
| 14. Delta (concentration) | -.030 | .321^**^ | .136^**^ | .608^**^ | .804^**^ | .216^**^ | .317^**^ | .198^**^ | .626^**^ | .847^**^ | .261^**^ | .530^**^ | .264^**^ | .663^**^ | 1 |  |
| 15. Spatial Proximity (clustering) | -.157^**^ | .338^**^ | .251^**^ | .252^**^ | .231^**^ | .329^**^ | .425^**^ | .395^**^ | .278^**^ | .267^**^ | .414^**^ | .729^**^ | .867^**^ | .247^**^ | .275^**^ | 1 |

**. Correlation is significant at the 0.01 level (2-tailed).
