# Supplementary material for: Death by Segregation: Does the Dimension of Racial Segregation Matter?
Source: PLoS One. 2015 Sep 23;10(9):e0138489. doi: 10.1371/journal.pone.0138489 (PMC4580431; doi:10.1371/journal.pone.0138489)
Supplement: S2 File — (DOCX) [file pone.0138489.s002.docx]

Figure A. Spatial Distribution of Mainline Protestants in US counties, by Quintiles (maps are created by the authors and the shapefiles are publicly available online).


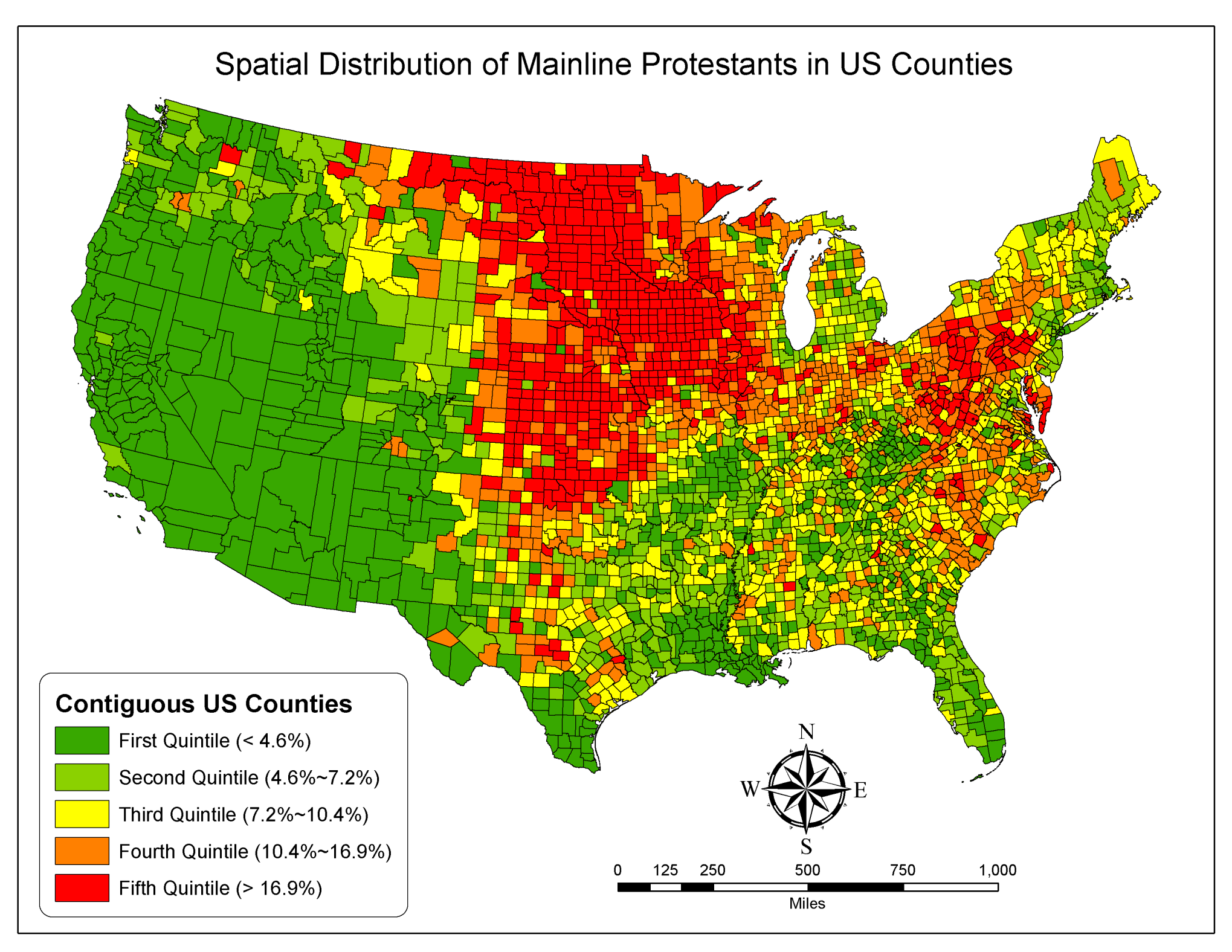


**Data source: Social Explorer Report (www.socialexplorer.com).**
